# 1-[(Ferrocen-1-yl)meth­yl]-3-(naphthalen-1-yl)thio­urea

**DOI:** 10.1107/S1600536811046629

**Published:** 2011-11-12

**Authors:** Xia Li, Wei Liu

**Affiliations:** a Department of Chemistry and Chemical Engineering, Henan University of Urban Construction, Pingdingshan, Henan 467044, People’s Republic of China

## Abstract

In the title compound, [Fe(C_5_H_5_)(C_17_H_15_N_2_S)], the cyclo­penta­dienyl (Cp) rings are almost parallel and essentially eclipsed, with a dihedral angle between the Cp ring planes of 0.807 (11)°. The Fe atom is slightly closer to the substituted cyclo­penta­dienyl ring, with an Fe–centroid distance of 1.6510 (8) Å, compared with 1.6597 (8) Å for the unsubstituted ring. The bridging unit between the substituted Cp ring and the naphthyl ring system is planar within 0.0174 Å and makes dihedral angles of 59.032 (10) and 66.02 (2)°, respectively, with these two rings. The angle between the substituted Cp ring and the naphthyl ring system is 72.094 (18)°. The H atoms of the NH groups of the thio­urea moiety are positioned *anti* with respect to each other. In the crystal, mol­ecules form centrosymmetric dimers *via* pairs of N—H⋯S hydrogen bonds.

## Related literature

For applications of thio­urea in the field of medicine, see: Di Grandi *et al.* (2004 ▶); Suh *et al.* (2005 ▶); Kaymakcioglu *et al.* (2005 ▶); Han *et al.* (2006 ▶), in bioorganic chemistry, see: Rostom (2006 ▶) and in supra­molecular chemistry, see: Henderson *et al.* (2001 ▶); Heck & Marsura (2003 ▶).
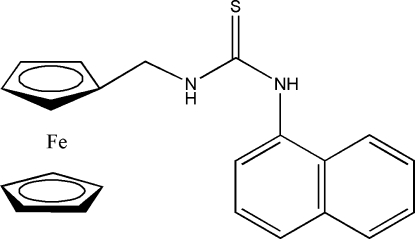


## Experimental

### 

#### Crystal data

[Fe(C_5_H_5_)(C_17_H_15_N_2_S)]
*M* *_r_* = 400.31
Triclinic, 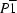

*a* = 7.958 (3) Å
*b* = 10.890 (5) Å
*c* = 12.357 (5) Å
α = 66.886 (6)°
β = 78.637 (8)°
γ = 73.306 (8)°
*V* = 939.1 (7) Å^3^
*Z* = 2
Mo *K*α radiation
μ = 0.92 mm^−1^
*T* = 296 K
0.39 × 0.24 × 0.16 mm

#### Data collection

Bruker SMART CCD area-detector diffractometer
Absorption correction: multi-scan (*SADABS*; Sheldrick, 2001 ▶) *T*_min_ = 0.755, *T*_max_ = 0.867
5178 measured reflections
3645 independent reflections
2741 reflections with *I* > 2σ(*I*)
*R*_int_ = 0.018

#### Refinement

*R*[*F* ^2^ > 2σ(*F* ^2^)] = 0.040
*wR*(*F* ^2^) = 0.105
*S* = 1.04
3645 reflections
241 parameters
H atoms treated by a mixture of independent and constrained refinement
Δρ_max_ = 0.35 e Å^−3^
Δρ_min_ = −0.32 e Å^−3^

### 

Data collection: *SMART* (Bruker, 2001 ▶); cell refinement: *SAINT* (Bruker, 2001 ▶); data reduction: *SAINT*; program(s) used to solve structure: *SHELXS97* (Sheldrick, 2008 ▶); program(s) used to refine structure: *SHELXL97* (Sheldrick, 2008 ▶); molecular graphics: *SHELXTL* (Sheldrick, 2008 ▶); software used to prepare material for publication: *SHELXTL*.

## Supplementary Material

Crystal structure: contains datablock(s) I, global. DOI: 10.1107/S1600536811046629/fj2464sup1.cif

Structure factors: contains datablock(s) I. DOI: 10.1107/S1600536811046629/fj2464Isup2.hkl

Additional supplementary materials:  crystallographic information; 3D view; checkCIF report

## Figures and Tables

**Table 1:** Hydrogen-bond geometry (Å, °)

*D*—H⋯*A*	*D*—H	H⋯*A*	*D*⋯*A*	*D*—H⋯*A*
N2—H2*A*⋯S1^i^	0.90 (3)	2.45 (3)	3.326 (3)	167 (2)

Symmetry code: (i) 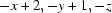.

**Fig. 1.**
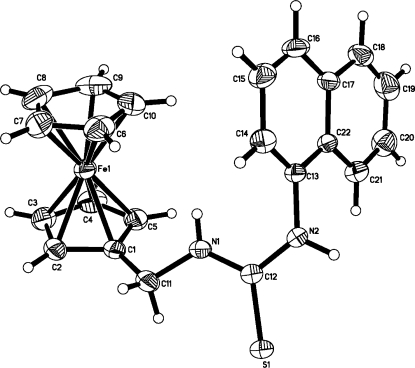
The molecular structure of the title compound, showing the atomic numbering and 30% probability displacement ellipsoids.

## supplementary crystallographic information

### Comment

Thiourea and its derivatives have attracted great attention because of their potential applications in the field of medicine (Di Grandi *et al.*, 2004; Suh *et al.*, 2005; Kaymakcioglu *et al.*, 2005; Han *et al.*, 2006), bioorganic (Rostom *et al.*, 2006) and supramolecular chemistry (Henderson *et al.*, 2001; Heck *et al.*, 2003). Detailed information on their molecular and crystal structures is necessary to understand their biologic activity and coordination possibility. Here we want to report the crystal structure of a new ferrocene-containing thiourea, 1-((ferroecen-1-yl)methyl)-3-(naphthalen-1-yl)thiourea.

The molecular structure of the title compound is composed of a (ferroecen-1-yl)methyl group and a naphthalen group joined by an organic thiourea spacer. The Fe—C bond distances within the ferrocene group are in the range of 2.043 (3)–2.048 (3) Å for the substituted cyclopentadienyl (Cp) ring [C1—C5] and 2.033 (3)–2.048 (3) Å for the unsubstituted Cp ring [C6—C10]. The Cp rings are almost parallel and are essentially eclipsed, and the dihedral angle between the Cp ring planes is 0.807 (11) °. The Fe atom is slightly closer to the substituted cyclopentadienyl ring, with a Fe-centroid distance of 1.6510 (8) Å, compared with 1.6597 (8) Å for the unsubstituted ring. The bridging unit between the substituted Cp ring and naphthyl rings is planar within 0.0174 Å and makes dihedral angles of 59.032 (10) ° and 66.019 (21) °, respectively, with these two rings, while the angle between the substituted Cp ring and naphthyl rings is 72.094 (18) °. The H atoms of the NH groups of thiourea are positioned anti to each other. In the crystal, the molecules form centrosymmetric dimers *via* intermolecular N—H···S hydrogen bonds.

### Experimental

To a solution of (ferrocene-1-yl)methanamine (1.075 g, 5 mmol) in MeOH (30 ml), 1-naphthyl isothiocyanate (0.925 g, 5 mmol) was added. The reaction mixture was stirred at room temperature for 12 h. The resulting solution was concentrated to about 10 ml and then cooled at ice-bath. The yellow precipitate was collected by filtration and washed with Ether several times. The crude product was purified by recrystallization from CH_2_Cl_2_ / MeOH to give 1-((ferroecen-1-yl)methyl)-3-(naphthalen-1-yl)thiourea as yellow block crystals.

### Refinement

H atoms on both the N and C atoms were positioned geometrically with N—H = 0.86 Å, C—H = 0.93 and 0.97 Å for aromatic and methyl H, and constrained to ride on their parent atoms with *U*_iso_(H)= 1.2Ueq(parent atom).

### Crystal data

**Table:**

[Fe(C_5_H_5_)(C_17_H_15_N_2_S)]	*V* = 939.1 (7) Å^3^
*M**_r_* = 400.31	*Z* = 2
Triclinic, *P*1	*F*(000) = 416
Hall symbol: -P 1	*D*_x_ = 1.416 Mg m^−^^3^
*a* = 7.958 (3) Å	Mo *K*α radiation, λ = 0.71073 Å
*b* = 10.890 (5) Å	θ = 1.8–28.2°
*c* = 12.357 (5) Å	µ = 0.92 mm^−^^1^
α = 66.886 (6)°	*T* = 296 K
β = 78.637 (8)°	Block, orange
γ = 73.306 (8)°	0.39 × 0.24 × 0.16 mm

### Data collection

**Table:**

Bruker SMART CCD area-detector diffractometer	3645 independent reflections
Radiation source: fine-focus sealed tube	2741 reflections with *I* > 2σ(*I*)
graphite	*R*_int_ = 0.018
phi and ω scans	θ_max_ = 26.0°, θ_min_ = 1.8°
Absorption correction: multi-scan (*SADABS*; Sheldrick, 2001)	*h* = −9→9
*T*_min_ = 0.755, *T*_max_ = 0.867	*k* = −9→13
5178 measured reflections	*l* = −14→15

### Refinement

**Table:**

Refinement on *F*^2^	Primary atom site location: structure-invariant direct methods
Least-squares matrix: full	Secondary atom site location: difference Fourier map
*R*[*F*^2^ > 2σ(*F*^2^)] = 0.040	Hydrogen site location: inferred from neighbouring sites
*wR*(*F*^2^) = 0.105	H atoms treated by a mixture of independent and constrained refinement
*S* = 1.04	*w* = 1/[σ^2^(*F*_o_^2^) + (0.0467*P*)^2^ + 0.1883*P*] where *P* = (*F*_o_^2^ + 2*F*_c_^2^)/3
3645 reflections	(Δ/σ)_max_ < 0.001
241 parameters	Δρ_max_ = 0.35 e Å^−^^3^
0 restraints	Δρ_min_ = −0.32 e Å^−^^3^

### Special details

**Table:**

Geometry. All e.s.d.'s (except the e.s.d. in the dihedral angle between two l.s. planes) are estimated using the full covariance matrix. The cell e.s.d.'s are taken into account individually in the estimation of e.s.d.'s in distances, angles and torsion angles; correlations between e.s.d.'s in cell parameters are only used when they are defined by crystal symmetry. An approximate (isotropic) treatment of cell e.s.d.'s is used for estimating e.s.d.'s involving l.s. planes.
Refinement. Refinement of *F*^2^ against ALL reflections. The weighted *R*-factor *wR* and goodness of fit *S* are based on *F*^2^, conventional *R*-factors *R* are based on *F*, with *F* set to zero for negative *F*^2^. The threshold expression of *F*^2^ > σ(*F*^2^) is used only for calculating *R*-factors(gt) *etc*. and is not relevant to the choice of reflections for refinement. *R*-factors based on *F*^2^ are statistically about twice as large as those based on *F*, and *R*- factors based on ALL data will be even larger.

### Fractional atomic coordinates and isotropic or equivalent isotropic displacement parameters (Å^2^)

**Table:**

	*x*	*y*	*z*	*U*_iso_*/*U*_eq_	
Fe1	0.28952 (5)	0.28154 (4)	0.44361 (3)	0.04797 (15)	
S1	0.81798 (10)	0.59967 (7)	0.11000 (7)	0.0553 (2)	
N1	0.6093 (3)	0.4257 (2)	0.2183 (2)	0.0559 (7)	
N2	0.8196 (3)	0.3739 (2)	0.0768 (2)	0.0513 (6)	
C1	0.4345 (4)	0.4236 (3)	0.4046 (2)	0.0500 (7)	
C2	0.2684 (4)	0.4629 (3)	0.4647 (3)	0.0558 (7)	
H2	0.1844	0.5443	0.4358	0.067*	
C3	0.2523 (5)	0.3575 (3)	0.5759 (3)	0.0645 (8)	
H3	0.1556	0.3570	0.6322	0.077*	
C4	0.4083 (5)	0.2533 (4)	0.5866 (3)	0.0689 (9)	
H4	0.4326	0.1721	0.6512	0.083*	
C5	0.5222 (4)	0.2936 (3)	0.4814 (3)	0.0602 (8)	
H5	0.6343	0.2438	0.4655	0.072*	
C6	0.2304 (5)	0.2825 (4)	0.2899 (3)	0.0716 (9)	
H6	0.2558	0.3436	0.2146	0.086*	
C7	0.0759 (4)	0.3020 (4)	0.3636 (3)	0.0747 (10)	
H7	−0.0190	0.3778	0.3458	0.090*	
C8	0.0883 (6)	0.1880 (5)	0.4690 (4)	0.0851 (12)	
H8	0.0040	0.1739	0.5338	0.102*	
C9	0.2543 (6)	0.0979 (4)	0.4579 (4)	0.0871 (12)	
H9	0.2987	0.0137	0.5147	0.104*	
C10	0.3395 (5)	0.1579 (4)	0.3468 (4)	0.0780 (10)	
H10	0.4501	0.1204	0.3166	0.094*	
C11	0.5046 (4)	0.5097 (3)	0.2864 (3)	0.0634 (8)	
H11A	0.4071	0.5746	0.2429	0.076*	
H11B	0.5770	0.5615	0.2968	0.076*	
C12	0.7436 (3)	0.4578 (3)	0.1373 (2)	0.0433 (6)	
C13	0.7688 (3)	0.2533 (3)	0.0865 (2)	0.0445 (6)	
C14	0.6119 (4)	0.2638 (3)	0.0504 (3)	0.0550 (7)	
H14	0.5362	0.3497	0.0215	0.066*	
C15	0.5630 (4)	0.1469 (4)	0.0563 (3)	0.0646 (9)	
H15	0.4541	0.1553	0.0340	0.078*	
C16	0.6750 (4)	0.0220 (4)	0.0946 (3)	0.0623 (8)	
H16	0.6429	−0.0546	0.0968	0.075*	
C17	0.8410 (4)	0.0057 (3)	0.1316 (2)	0.0492 (7)	
C18	0.9628 (5)	−0.1220 (3)	0.1691 (3)	0.0666 (9)	
H18	0.9360	−0.1994	0.1686	0.080*	
C19	1.1183 (5)	−0.1343 (3)	0.2060 (3)	0.0779 (10)	
H19	1.1966	−0.2198	0.2303	0.093*	
C20	1.1618 (4)	−0.0202 (4)	0.2079 (3)	0.0700 (9)	
H20	1.2678	−0.0305	0.2351	0.084*	
C21	1.0513 (4)	0.1059 (3)	0.1705 (3)	0.0562 (7)	
H21	1.0828	0.1814	0.1714	0.067*	
C22	0.8878 (3)	0.1236 (3)	0.1299 (2)	0.0426 (6)	
H1A	0.577 (4)	0.357 (3)	0.230 (2)	0.051*	
H2A	0.921 (4)	0.389 (3)	0.033 (2)	0.051*	

### Atomic displacement parameters (Å^2^)

**Table:**

	*U*^11^	*U*^22^	*U*^33^	*U*^12^	*U*^13^	*U*^23^
Fe1	0.0496 (2)	0.0505 (3)	0.0520 (3)	−0.01930 (18)	−0.00125 (18)	−0.02317 (19)
S1	0.0595 (4)	0.0442 (4)	0.0712 (5)	−0.0267 (3)	0.0129 (4)	−0.0287 (4)
N1	0.0636 (15)	0.0482 (14)	0.0679 (16)	−0.0311 (12)	0.0227 (12)	−0.0341 (13)
N2	0.0472 (13)	0.0477 (14)	0.0668 (16)	−0.0240 (11)	0.0165 (11)	−0.0298 (12)
C1	0.0560 (16)	0.0482 (16)	0.0578 (17)	−0.0207 (13)	0.0047 (13)	−0.0301 (14)
C2	0.0613 (18)	0.0536 (17)	0.0584 (18)	−0.0166 (14)	0.0067 (14)	−0.0298 (15)
C3	0.074 (2)	0.076 (2)	0.0555 (19)	−0.0343 (18)	0.0120 (16)	−0.0334 (17)
C4	0.091 (3)	0.072 (2)	0.0521 (19)	−0.0320 (19)	−0.0182 (18)	−0.0162 (17)
C5	0.0537 (17)	0.063 (2)	0.078 (2)	−0.0135 (14)	−0.0135 (16)	−0.0362 (17)
C6	0.083 (2)	0.090 (3)	0.063 (2)	−0.039 (2)	−0.0065 (19)	−0.036 (2)
C7	0.058 (2)	0.089 (3)	0.096 (3)	−0.0177 (18)	−0.0148 (19)	−0.049 (2)
C8	0.087 (3)	0.119 (3)	0.081 (3)	−0.069 (3)	0.015 (2)	−0.048 (3)
C9	0.113 (3)	0.055 (2)	0.107 (3)	−0.035 (2)	−0.040 (3)	−0.019 (2)
C10	0.076 (2)	0.086 (3)	0.104 (3)	−0.026 (2)	−0.004 (2)	−0.064 (3)
C11	0.076 (2)	0.0511 (18)	0.070 (2)	−0.0252 (15)	0.0233 (16)	−0.0351 (16)
C12	0.0441 (14)	0.0395 (14)	0.0496 (15)	−0.0149 (11)	−0.0004 (12)	−0.0178 (12)
C13	0.0486 (15)	0.0469 (15)	0.0494 (15)	−0.0234 (12)	0.0082 (12)	−0.0267 (13)
C14	0.0532 (17)	0.0577 (18)	0.0621 (18)	−0.0174 (14)	−0.0050 (14)	−0.0269 (15)
C15	0.0611 (19)	0.088 (3)	0.068 (2)	−0.0358 (18)	0.0002 (16)	−0.0422 (19)
C16	0.079 (2)	0.072 (2)	0.0627 (19)	−0.0482 (18)	0.0104 (16)	−0.0378 (17)
C17	0.0658 (18)	0.0450 (16)	0.0458 (15)	−0.0278 (14)	0.0115 (13)	−0.0232 (13)
C18	0.097 (3)	0.0452 (18)	0.0589 (19)	−0.0302 (17)	0.0167 (18)	−0.0212 (15)
C19	0.090 (3)	0.0484 (19)	0.074 (2)	−0.0020 (18)	0.003 (2)	−0.0143 (17)
C20	0.0595 (19)	0.067 (2)	0.075 (2)	−0.0083 (16)	−0.0095 (17)	−0.0198 (18)
C21	0.0570 (17)	0.0568 (18)	0.0623 (18)	−0.0237 (14)	0.0004 (14)	−0.0247 (15)
C22	0.0479 (15)	0.0470 (15)	0.0425 (14)	−0.0238 (12)	0.0093 (12)	−0.0233 (12)

### Geometric parameters (Å, °)

**Table:**

Fe1—C9	2.033 (3)	C6—H6	0.9300
Fe1—C6	2.040 (3)	C7—C8	1.400 (5)
Fe1—C10	2.042 (3)	C7—H7	0.9300
Fe1—C4	2.043 (3)	C8—C9	1.420 (6)
Fe1—C3	2.043 (3)	C8—H8	0.9300
Fe1—C1	2.044 (3)	C9—C10	1.398 (5)
Fe1—C7	2.046 (3)	C9—H9	0.9300
Fe1—C8	2.047 (3)	C10—H10	0.9300
Fe1—C5	2.047 (3)	C11—H11A	0.9700
Fe1—C2	2.048 (3)	C11—H11B	0.9700
S1—C12	1.699 (3)	C13—C14	1.366 (4)
N1—C12	1.336 (3)	C13—C22	1.420 (4)
N1—C11	1.458 (3)	C14—C15	1.407 (4)
N1—H1A	0.81 (3)	C14—H14	0.9300
N2—C12	1.342 (3)	C15—C16	1.354 (5)
N2—C13	1.437 (3)	C15—H15	0.9300
N2—H2A	0.90 (3)	C16—C17	1.423 (4)
C1—C2	1.421 (4)	C16—H16	0.9300
C1—C5	1.425 (4)	C17—C18	1.409 (4)
C1—C11	1.493 (4)	C17—C22	1.428 (3)
C2—C3	1.414 (4)	C18—C19	1.355 (5)
C2—H2	0.9300	C18—H18	0.9300
C3—C4	1.409 (5)	C19—C20	1.393 (5)
C3—H3	0.9300	C19—H19	0.9300
C4—C5	1.421 (4)	C20—C21	1.357 (4)
C4—H4	0.9300	C20—H20	0.9300
C5—H5	0.9300	C21—C22	1.421 (4)
C6—C10	1.380 (5)	C21—H21	0.9300
C6—C7	1.396 (5)		
			
C9—Fe1—C6	66.95 (16)	C4—C5—C1	108.0 (3)
C9—Fe1—C10	40.13 (16)	C4—C5—Fe1	69.49 (17)
C6—Fe1—C10	39.51 (14)	C1—C5—Fe1	69.49 (16)
C9—Fe1—C4	108.65 (15)	C4—C5—H5	126.0
C6—Fe1—C4	165.75 (15)	C1—C5—H5	126.0
C10—Fe1—C4	128.50 (16)	Fe1—C5—H5	126.6
C9—Fe1—C3	127.08 (16)	C10—C6—C7	108.9 (3)
C6—Fe1—C3	153.19 (15)	C10—C6—Fe1	70.3 (2)
C10—Fe1—C3	165.09 (16)	C7—C6—Fe1	70.26 (19)
C4—Fe1—C3	40.33 (13)	C10—C6—H6	125.6
C9—Fe1—C1	154.07 (16)	C7—C6—H6	125.6
C6—Fe1—C1	109.02 (13)	Fe1—C6—H6	125.4
C10—Fe1—C1	120.19 (14)	C6—C7—C8	108.3 (4)
C4—Fe1—C1	68.58 (12)	C6—C7—Fe1	69.80 (19)
C3—Fe1—C1	68.53 (12)	C8—C7—Fe1	70.0 (2)
C9—Fe1—C7	67.36 (16)	C6—C7—H7	125.8
C6—Fe1—C7	39.94 (13)	C8—C7—H7	125.8
C10—Fe1—C7	67.04 (15)	Fe1—C7—H7	125.9
C4—Fe1—C7	152.63 (15)	C7—C8—C9	106.7 (3)
C3—Fe1—C7	118.87 (14)	C7—C8—Fe1	69.98 (19)
C1—Fe1—C7	127.19 (14)	C9—C8—Fe1	69.1 (2)
C9—Fe1—C8	40.73 (16)	C7—C8—H8	126.6
C6—Fe1—C8	67.34 (15)	C9—C8—H8	126.6
C10—Fe1—C8	67.84 (15)	Fe1—C8—H8	125.8
C4—Fe1—C8	118.94 (15)	C10—C9—C8	108.1 (4)
C3—Fe1—C8	107.16 (14)	C10—C9—Fe1	70.29 (19)
C1—Fe1—C8	163.82 (16)	C8—C9—Fe1	70.2 (2)
C7—Fe1—C8	39.99 (15)	C10—C9—H9	125.9
C9—Fe1—C5	120.16 (15)	C8—C9—H9	125.9
C6—Fe1—C5	128.50 (13)	Fe1—C9—H9	125.2
C10—Fe1—C5	109.68 (14)	C6—C10—C9	108.0 (4)
C4—Fe1—C5	40.66 (13)	C6—C10—Fe1	70.17 (19)
C3—Fe1—C5	68.15 (13)	C9—C10—Fe1	69.6 (2)
C1—Fe1—C5	40.76 (12)	C6—C10—H10	126.0
C7—Fe1—C5	165.29 (15)	C9—C10—H10	126.0
C8—Fe1—C5	153.72 (17)	Fe1—C10—H10	125.8
C9—Fe1—C2	164.22 (17)	N1—C11—C1	111.5 (2)
C6—Fe1—C2	120.10 (14)	N1—C11—H11A	109.3
C10—Fe1—C2	153.81 (15)	C1—C11—H11A	109.3
C4—Fe1—C2	67.90 (13)	N1—C11—H11B	109.3
C3—Fe1—C2	40.44 (12)	C1—C11—H11B	109.3
C1—Fe1—C2	40.65 (11)	H11A—C11—H11B	108.0
C7—Fe1—C2	108.15 (14)	N1—C12—N2	117.7 (2)
C8—Fe1—C2	126.17 (15)	N1—C12—S1	122.01 (19)
C5—Fe1—C2	68.03 (12)	N2—C12—S1	120.24 (19)
C12—N1—C11	125.0 (2)	C14—C13—C22	120.7 (2)
C12—N1—H1A	120 (2)	C14—C13—N2	120.6 (3)
C11—N1—H1A	115 (2)	C22—C13—N2	118.7 (2)
C12—N2—C13	127.0 (2)	C13—C14—C15	121.1 (3)
C12—N2—H2A	115.8 (17)	C13—C14—H14	119.4
C13—N2—H2A	116.7 (17)	C15—C14—H14	119.4
C2—C1—C5	107.2 (3)	C16—C15—C14	119.8 (3)
C2—C1—C11	125.1 (3)	C16—C15—H15	120.1
C5—C1—C11	127.6 (3)	C14—C15—H15	120.1
C2—C1—Fe1	69.82 (16)	C15—C16—C17	121.3 (3)
C5—C1—Fe1	69.75 (16)	C15—C16—H16	119.4
C11—C1—Fe1	128.7 (2)	C17—C16—H16	119.4
C3—C2—C1	108.5 (3)	C18—C17—C16	122.9 (3)
C3—C2—Fe1	69.61 (17)	C18—C17—C22	118.2 (3)
C1—C2—Fe1	69.53 (15)	C16—C17—C22	119.0 (3)
C3—C2—H2	125.7	C19—C18—C17	121.4 (3)
C1—C2—H2	125.7	C19—C18—H18	119.3
Fe1—C2—H2	126.7	C17—C18—H18	119.3
C4—C3—C2	108.1 (3)	C18—C19—C20	120.5 (3)
C4—C3—Fe1	69.82 (18)	C18—C19—H19	119.7
C2—C3—Fe1	69.96 (16)	C20—C19—H19	119.7
C4—C3—H3	126.0	C21—C20—C19	120.7 (3)
C2—C3—H3	126.0	C21—C20—H20	119.6
Fe1—C3—H3	125.8	C19—C20—H20	119.6
C3—C4—C5	108.2 (3)	C20—C21—C22	120.5 (3)
C3—C4—Fe1	69.85 (18)	C20—C21—H21	119.7
C5—C4—Fe1	69.85 (17)	C22—C21—H21	119.7
C3—C4—H4	125.9	C13—C22—C21	123.2 (2)
C5—C4—H4	125.9	C13—C22—C17	118.1 (2)
Fe1—C4—H4	126.0	C21—C22—C17	118.6 (3)
			
C9—Fe1—C1—C2	−169.4 (3)	C10—Fe1—C6—C7	119.6 (3)
C6—Fe1—C1—C2	114.4 (2)	C4—Fe1—C6—C7	156.5 (5)
C10—Fe1—C1—C2	156.4 (2)	C3—Fe1—C6—C7	−45.9 (4)
C4—Fe1—C1—C2	−80.6 (2)	C1—Fe1—C6—C7	−125.7 (2)
C3—Fe1—C1—C2	−37.12 (19)	C8—Fe1—C6—C7	37.4 (2)
C7—Fe1—C1—C2	73.5 (2)	C5—Fe1—C6—C7	−167.2 (2)
C8—Fe1—C1—C2	40.3 (5)	C2—Fe1—C6—C7	−82.4 (2)
C5—Fe1—C1—C2	−118.1 (2)	C10—C6—C7—C8	0.3 (4)
C9—Fe1—C1—C5	−51.2 (4)	Fe1—C6—C7—C8	−59.7 (2)
C6—Fe1—C1—C5	−127.48 (19)	C10—C6—C7—Fe1	59.9 (2)
C10—Fe1—C1—C5	−85.5 (2)	C9—Fe1—C7—C6	−80.6 (3)
C4—Fe1—C1—C5	37.54 (18)	C10—Fe1—C7—C6	−36.9 (2)
C3—Fe1—C1—C5	81.0 (2)	C4—Fe1—C7—C6	−167.7 (3)
C7—Fe1—C1—C5	−168.33 (19)	C3—Fe1—C7—C6	158.3 (2)
C8—Fe1—C1—C5	158.4 (4)	C1—Fe1—C7—C6	74.4 (3)
C2—Fe1—C1—C5	118.1 (2)	C8—Fe1—C7—C6	−119.3 (3)
C9—Fe1—C1—C11	71.3 (4)	C5—Fe1—C7—C6	43.1 (6)
C6—Fe1—C1—C11	−4.9 (3)	C2—Fe1—C7—C6	115.5 (2)
C10—Fe1—C1—C11	37.0 (3)	C9—Fe1—C7—C8	38.7 (2)
C4—Fe1—C1—C11	160.1 (3)	C6—Fe1—C7—C8	119.3 (3)
C3—Fe1—C1—C11	−156.4 (3)	C10—Fe1—C7—C8	82.4 (3)
C7—Fe1—C1—C11	−45.8 (3)	C4—Fe1—C7—C8	−48.3 (4)
C8—Fe1—C1—C11	−79.0 (5)	C3—Fe1—C7—C8	−82.4 (3)
C5—Fe1—C1—C11	122.5 (3)	C1—Fe1—C7—C8	−166.3 (2)
C2—Fe1—C1—C11	−119.3 (3)	C5—Fe1—C7—C8	162.4 (5)
C5—C1—C2—C3	−1.2 (3)	C2—Fe1—C7—C8	−125.1 (2)
C11—C1—C2—C3	−177.4 (3)	C6—C7—C8—C9	−0.1 (4)
Fe1—C1—C2—C3	58.9 (2)	Fe1—C7—C8—C9	−59.6 (2)
C5—C1—C2—Fe1	−60.01 (18)	C6—C7—C8—Fe1	59.5 (2)
C11—C1—C2—Fe1	123.7 (3)	C9—Fe1—C8—C7	−117.8 (3)
C9—Fe1—C2—C3	42.7 (6)	C6—Fe1—C8—C7	−37.3 (2)
C6—Fe1—C2—C3	155.5 (2)	C10—Fe1—C8—C7	−80.2 (2)
C10—Fe1—C2—C3	−171.8 (3)	C4—Fe1—C8—C7	156.9 (2)
C4—Fe1—C2—C3	−37.6 (2)	C3—Fe1—C8—C7	114.7 (2)
C1—Fe1—C2—C3	−120.0 (3)	C1—Fe1—C8—C7	42.8 (6)
C7—Fe1—C2—C3	113.5 (2)	C5—Fe1—C8—C7	−170.0 (3)
C8—Fe1—C2—C3	72.9 (3)	C2—Fe1—C8—C7	74.3 (3)
C5—Fe1—C2—C3	−81.6 (2)	C6—Fe1—C8—C9	80.5 (3)
C9—Fe1—C2—C1	162.8 (5)	C10—Fe1—C8—C9	37.6 (2)
C6—Fe1—C2—C1	−84.4 (2)	C4—Fe1—C8—C9	−85.3 (3)
C10—Fe1—C2—C1	−51.8 (4)	C3—Fe1—C8—C9	−127.5 (2)
C4—Fe1—C2—C1	82.4 (2)	C1—Fe1—C8—C9	160.6 (4)
C3—Fe1—C2—C1	120.0 (3)	C7—Fe1—C8—C9	117.8 (3)
C7—Fe1—C2—C1	−126.5 (2)	C5—Fe1—C8—C9	−52.2 (4)
C8—Fe1—C2—C1	−167.1 (2)	C2—Fe1—C8—C9	−167.9 (2)
C5—Fe1—C2—C1	38.38 (18)	C7—C8—C9—C10	−0.1 (4)
C1—C2—C3—C4	0.8 (3)	Fe1—C8—C9—C10	−60.3 (2)
Fe1—C2—C3—C4	59.6 (2)	C7—C8—C9—Fe1	60.2 (2)
C1—C2—C3—Fe1	−58.80 (19)	C6—Fe1—C9—C10	37.2 (2)
C9—Fe1—C3—C4	74.3 (3)	C4—Fe1—C9—C10	−128.2 (2)
C6—Fe1—C3—C4	−171.7 (3)	C3—Fe1—C9—C10	−169.4 (2)
C10—Fe1—C3—C4	46.7 (6)	C1—Fe1—C9—C10	−49.0 (4)
C1—Fe1—C3—C4	−81.8 (2)	C7—Fe1—C9—C10	80.7 (2)
C7—Fe1—C3—C4	156.6 (2)	C8—Fe1—C9—C10	118.7 (3)
C8—Fe1—C3—C4	114.8 (2)	C5—Fe1—C9—C10	−85.1 (3)
C5—Fe1—C3—C4	−37.76 (19)	C2—Fe1—C9—C10	157.2 (5)
C2—Fe1—C3—C4	−119.1 (3)	C6—Fe1—C9—C8	−81.5 (3)
C9—Fe1—C3—C2	−166.6 (2)	C10—Fe1—C9—C8	−118.7 (3)
C6—Fe1—C3—C2	−52.6 (4)	C4—Fe1—C9—C8	113.0 (3)
C10—Fe1—C3—C2	165.8 (5)	C3—Fe1—C9—C8	71.9 (3)
C4—Fe1—C3—C2	119.1 (3)	C1—Fe1—C9—C8	−167.8 (3)
C1—Fe1—C3—C2	37.31 (18)	C7—Fe1—C9—C8	−38.0 (2)
C7—Fe1—C3—C2	−84.3 (2)	C5—Fe1—C9—C8	156.1 (2)
C8—Fe1—C3—C2	−126.2 (2)	C2—Fe1—C9—C8	38.4 (6)
C5—Fe1—C3—C2	81.3 (2)	C7—C6—C10—C9	−0.4 (4)
C2—C3—C4—C5	−0.2 (3)	Fe1—C6—C10—C9	59.6 (2)
Fe1—C3—C4—C5	59.5 (2)	C7—C6—C10—Fe1	−59.9 (2)
C2—C3—C4—Fe1	−59.7 (2)	C8—C9—C10—C6	0.3 (4)
C9—Fe1—C4—C3	−125.8 (2)	Fe1—C9—C10—C6	−59.9 (2)
C6—Fe1—C4—C3	164.6 (5)	C8—C9—C10—Fe1	60.2 (2)
C10—Fe1—C4—C3	−166.1 (2)	C9—Fe1—C10—C6	118.9 (3)
C1—Fe1—C4—C3	81.6 (2)	C4—Fe1—C10—C6	−169.1 (2)
C7—Fe1—C4—C3	−49.2 (4)	C3—Fe1—C10—C6	153.9 (5)
C8—Fe1—C4—C3	−82.5 (2)	C1—Fe1—C10—C6	−83.5 (2)
C5—Fe1—C4—C3	119.3 (3)	C7—Fe1—C10—C6	37.3 (2)
C2—Fe1—C4—C3	37.71 (18)	C8—Fe1—C10—C6	80.8 (2)
C9—Fe1—C4—C5	114.9 (2)	C5—Fe1—C10—C6	−127.3 (2)
C6—Fe1—C4—C5	45.3 (6)	C2—Fe1—C10—C6	−47.2 (4)
C10—Fe1—C4—C5	74.6 (2)	C6—Fe1—C10—C9	−118.9 (3)
C3—Fe1—C4—C5	−119.3 (3)	C4—Fe1—C10—C9	72.0 (3)
C1—Fe1—C4—C5	−37.63 (18)	C3—Fe1—C10—C9	34.9 (6)
C7—Fe1—C4—C5	−168.5 (3)	C1—Fe1—C10—C9	157.5 (2)
C8—Fe1—C4—C5	158.2 (2)	C7—Fe1—C10—C9	−81.6 (3)
C2—Fe1—C4—C5	−81.55 (19)	C8—Fe1—C10—C9	−38.1 (2)
C3—C4—C5—C1	−0.5 (3)	C5—Fe1—C10—C9	113.8 (2)
Fe1—C4—C5—C1	59.01 (19)	C2—Fe1—C10—C9	−166.2 (3)
C3—C4—C5—Fe1	−59.5 (2)	C12—N1—C11—C1	−150.6 (3)
C2—C1—C5—C4	1.0 (3)	C2—C1—C11—N1	−145.3 (3)
C11—C1—C5—C4	177.2 (3)	C5—C1—C11—N1	39.2 (4)
Fe1—C1—C5—C4	−59.02 (19)	Fe1—C1—C11—N1	−54.1 (4)
C2—C1—C5—Fe1	60.05 (19)	C11—N1—C12—N2	−175.8 (3)
C11—C1—C5—Fe1	−123.8 (3)	C11—N1—C12—S1	4.0 (4)
C9—Fe1—C5—C4	−83.8 (2)	C13—N2—C12—N1	2.1 (4)
C6—Fe1—C5—C4	−167.1 (2)	C13—N2—C12—S1	−177.7 (2)
C10—Fe1—C5—C4	−126.8 (2)	C12—N2—C13—C14	67.5 (4)
C3—Fe1—C5—C4	37.46 (19)	C12—N2—C13—C22	−115.0 (3)
C1—Fe1—C5—C4	119.5 (3)	C22—C13—C14—C15	0.7 (4)
C7—Fe1—C5—C4	158.9 (5)	N2—C13—C14—C15	178.1 (2)
C8—Fe1—C5—C4	−47.2 (4)	C13—C14—C15—C16	−2.2 (4)
C2—Fe1—C5—C4	81.2 (2)	C14—C15—C16—C17	1.3 (5)
C9—Fe1—C5—C1	156.8 (2)	C15—C16—C17—C18	−178.4 (3)
C6—Fe1—C5—C1	73.5 (2)	C15—C16—C17—C22	1.1 (4)
C10—Fe1—C5—C1	113.8 (2)	C16—C17—C18—C19	−178.7 (3)
C4—Fe1—C5—C1	−119.5 (3)	C22—C17—C18—C19	1.9 (4)
C3—Fe1—C5—C1	−82.01 (19)	C17—C18—C19—C20	0.1 (5)
C7—Fe1—C5—C1	39.4 (6)	C18—C19—C20—C21	−1.4 (5)
C8—Fe1—C5—C1	−166.6 (3)	C19—C20—C21—C22	0.7 (5)
C2—Fe1—C5—C1	−38.27 (16)	C14—C13—C22—C21	−178.9 (2)
C9—Fe1—C6—C10	−37.8 (2)	N2—C13—C22—C21	3.6 (4)
C4—Fe1—C6—C10	36.9 (6)	C14—C13—C22—C17	1.7 (4)
C3—Fe1—C6—C10	−165.4 (3)	N2—C13—C22—C17	−175.8 (2)
C1—Fe1—C6—C10	114.7 (2)	C20—C21—C22—C13	−178.2 (3)
C7—Fe1—C6—C10	−119.6 (3)	C20—C21—C22—C17	1.2 (4)
C8—Fe1—C6—C10	−82.2 (3)	C18—C17—C22—C13	177.0 (2)
C5—Fe1—C6—C10	73.2 (3)	C16—C17—C22—C13	−2.5 (4)
C2—Fe1—C6—C10	158.0 (2)	C18—C17—C22—C21	−2.5 (4)
C9—Fe1—C6—C7	81.8 (3)	C16—C17—C22—C21	178.0 (2)

### Hydrogen-bond geometry (Å, °)

**Table:**

*D*—H···*A*	*D*—H	H···*A*	*D*···*A*	*D*—H···*A*
N2—H2A···S1^i^	0.90 (3)	2.45 (3)	3.326 (3)	167 (2)

Symmetry codes: (i) −*x*+2, −*y*+1, −*z*.
